# An Alternate Method to Springback Compensation for Sheet Metal Forming

**DOI:** 10.1155/2014/301271

**Published:** 2014-06-11

**Authors:** Waluyo Adi Siswanto, Agus Dwi Anggono, Badrul Omar, Kamaruzaman Jusoff

**Affiliations:** ^1^Department of Engineering Mechanics, Universiti Tun Hussein Onn Malaysia, Parit Raja, Batu Pahat, 86400 Johor, Malaysia; ^2^Department of Mechanical Engineering, Muhammadiyah University of Surakarta, Pabelan, Kartasura 57162, Indonesia; ^3^Department of Materials Engineering and Design, Universiti Tun Hussein Onn Malaysia, Parit Raja, 86400 Johor, Malaysia; ^4^Faculty of Forestry, Universiti Putra Malaysia, Serdang, 43400 Selangor, Malaysia

## Abstract

The aim of this work is to improve the accuracy of cold stamping product by accommodating springback. This is a numerical approach to improve the accuracy of springback analysis and die compensation process combining the displacement adjustment (DA) method and the spring forward (SF) algorithm. This alternate hybrid method (HM) is conducted by firstly employing DA method followed by the SF method instead of either DA or SF method individually. The springback shape and the target part are used to optimize the die surfaces compensating springback. The hybrid method (HM) algorithm has been coded in Fortran and tested in two- and three-dimensional models. By implementing the HM, the springback error can be decreased and the dimensional deviation falls in the predefined tolerance range.

## 1. Introduction

The Sheet metal forming (SMF) is one of forming processes performed on metal sheets, strips, and coils. Press working is the term often applied to sheet metal operations because the machines used to perform these operations are press machines. A part produced in a sheet metal operation is often called a stamping product. SMF process consists of stamping, forming, bending, stretching, and trimming. The terms refer to various processes used to convert a sheet metal into different shapes for a large variety of finished useful products. Since SMF process produces the same products, it has become one of the most important manufacturing processes in industry, particularly in the automotive and steel industries [1].

Every process of sheet metal forming or stamping process involves elastic forming and then followed by permanent plastic deformation. Since the presence of elastic properties of metals, after the unloading phase, the elastic springback phenomenon always occurs resulting in an off-target formed shape. Springback is dimensional deviation due to the elasticity of a metal sheet during unloading and following forming [2]. Although it is impossible to eliminate the springback phenomenon, minimizing springback can be done by adopting three approaches. The first one is based on controlling the blank holder force (BHF). The blank holding force is not fixed, but, as a controlled variable, the blank holding force is known as variable blank holder force (VBHF) so that the springback can be minimized. The second approach is by using a hot forming process and the third one is by optimizing die surfaces (die compensation) to accommodate the springback.

The VBHF was initially proposed by Liu et al. [3] to reduce the springback error of U-bend model of NUMISHEET′93. The VBHF was determined by calculating the value of low blank holder force (BHFL) and high blank holder force (BHFS). In the report [3], the VBHF approach successfully reduced the springback error compared with that from constant blank holder force (CBHF). The application of VBHF has been extended and published by researchers recently: Li et al. [4] investigated the subsection of VBHF in rectangular parts, and then Kitayama et al. [5] presented specifically the optimization of VBHF to reduce the springback. A precision binder force control during forming is required, making this process sensitive to any variations in manufacturing conditions such as punch speed, time control, and friction.

The study of springback reduction by the temperature value of tool in hot forming has been investigated [6]. It is found that the cold punch combined with the hot die can reduce the springback up to 20% when compared to conventional room temperature bending test in the sheet forming process of U-bend aluminum 1050. The experimental investigations in hot forming process can be found in the latest publications [7–9]. Temperature in the springback prediction is becoming a topic that could be improved in the future investigation [2]. The springback value before and after splitting process is different. This has been studied using split-ring tests for aluminum alloy [10].

The major advantage of the first strategy to minimize springback is that it is not necessary to modify the tooling surface. However, there are several implementation difficulties such as implementing the force control or force sensor in the application of VBHF being highly cost sensitive. Similarly, in the hot forming process, it requires additional equipment to control the temperatures of the tools and the blank sheet so that the forming process becomes more complicated and expensive.

In the third method (die compensation), it requires many steps of works on tooling design stage, but its potential to compensate springback completely is faster and cheaper even for complex model. Die face adjustment to compensate the springback was done manually in the past, by doing extensive measurements on the prototype or even production tools and refining the tool surface geometry by hand polishing which is time consuming [11]. Wagoner et al. [2] categorized the current research of springback in five topics, that is, plastic constitutive equations, variable Young's modulus, through-thickness integration of stress, magnesium, and advanced high strength steels (AHSS).

Finite element (FE) and numerical and process simulation have gained popularity in the stamping industry due to its speed and low cost, and it has been proven to be effective and efficient in the prediction of form ability and springback behavior [12, 13]. The accuracy of springback simulation is not only related to springback analysis itself but also strongly dependent on the accuracy of forming processes. Any calculation error obtained from every simulation step of forming processes will be accumulated. As a result, the accumulated error influence the accuracy of the springback prediction analysis at the last step of the simulation. Ling et al. [13] tried to reduce springback in L-bending by using the optimization of die radius, clearance, step height, and step distance. The results show a good reduction in springback, but this method is applied and tested in the L-bending only. The U-bending model is analyzed by Slota et al. [14] in both numerical and experimental analyses. The experimental analysis is conducted by using specimens of steel DC06, UHSS TRIP RAK 40/70, and HSS H220PD in orientations 0°, 45°, and 90°. The results show that the simulations are in a good agreement with the experiments. The springback in sheet forming can be simulated accurately by using finite element method, but the big problem is to apply the results to produce stamped parts in accurate dimension.

In order to accommodate the springback and to optimize the die based on surface modification, there are two methodologies available called displacement adjustment (DA) compensation and spring forward (SF) compensation. The concept of DA method [15] is to translate the nodes in the direction opposite to the springback which is adopted from the real springback investigation, whereas the SF method is based on stress state during forming, which is multiplied by negative factor and then loaded to the deformed shape to realize the compensated surface [16]. The compensation method based on calculated springback by implementing the DA method can be done in a computer program [17]. The modification of die surface for springback compensation can be done faster. There are many researchers who reported the implementation of these two methods in various springback accommodation problems and are dealing with minimizing springback errors [18–20].

One of the advantages of DA method is that it can converge rapidly because of the algorithm being based on the real springback measurements, not virtual springback calculation, which may develop instability during the numerical solution [15]. Unfortunately, the original DA method is mostly applicable to compensate surfaces in the direction of the punch travel. The wall area of the dies, therefore, could not be compensated [21].

On the other hand, the SF method could compensate die surface in almost all directions because of the deformation being caused by the stresses of the deformed part in the opposite direction [22]. In the original concept of spring forward [16], only inverted bending stress will be used to create the spring forward shape. The inverted membrane stresses must be eliminated since they will contribute to the unstable buckling condition during the numerical calculation. Another disadvantage of SF method is that the approach is not based on the reality of physical occurrence but an inverted springbok. The complexity of inverted bending stress distribution in the deformed shape is very difficult to apply in an experiment, even impossible [21]. In order to solve the problem, finite element simulation analysis can be used to explain the influence of residual stress in springback forming as done by Brabie et al. [23]. It shows that residual stress is influenced by the BHF and coefficient of friction in both cylindrical and conical forming. They analyze the residual stress during forming and after springback but do not explain how to minimize the springback related to the residual stress.

Force descriptor method (FDM) proposed by Karafillis and Boyce [22] is SF method based. This method has two parameter vectors, the original part shape and the internal forces. The theoretical base of the FDM is that after being formed to the original tooling shape, the part would springback to the original unloaded shape, recovering from the internal force. By spring forward, the stress free original shape with the reversed internal force, a tooling shape close to the original shape, could be obtained. Considering a simple bending case, the internal force in the first iteration is lower than the final force, which means that the FDM needed more iterations. Cheng et al. [21] modified this approach in the first iteration by replacing the internal force with the internal force obtained from the manual calculation to accelerate the compensation.

Wagoner et al. [24] proposed the displacement adjustment method. At first forming, a flat sheet is deformed to the original die shape. After springback, the shape is compared with its target to obtain the shape error. In the next step, the amount of shape error is added to the current die shape to obtain the new compensated shape. During the next iteration, a blank sheet is deformed to the new compensated shape. If the shape error is not in the range of tolerance, another iteration will be conducted. Wagoner and coworkers claimed this method is effective and converges more rapidly than the FDM.

In the present research, a new approach in accommodating springback is the main objective. This research aims at the development of a compensation procedure that can perform the optimization process, using the combination of fast convergence displacement adjustment (DA) and the flexible spring forward (SF) methods so that the combination method will be fast and applicable for all die surfaces. The method guides the die surface modification process to compensate for springback following the displacement adjustment; then it continues to compensate the springback error in forward direction (spring forward). The new combined method is then called hybrid method (HM) die compensation.

## 2. Proposed Compensation Method

**Figure 1:**
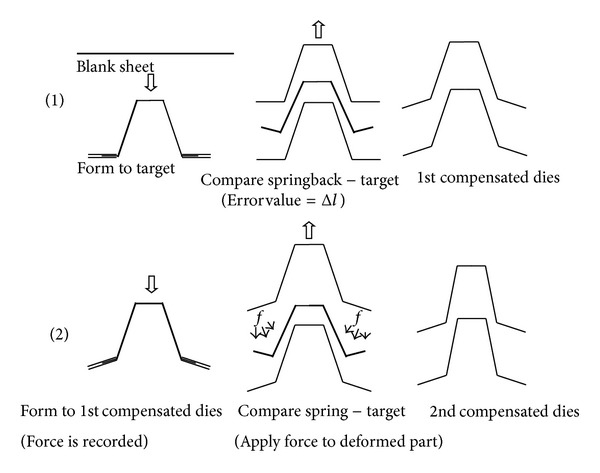
Illustration of compensation procedures in hybrid method.

The hybrid method (HM) is a combination of DA and SF method. The procedures of HM are illustrated in Figure 1. The basic process of the hybrid method can be explained as a simple forming process; a flat metal sheet is bent downwards beyond its elastic region.

The part is deformed into the initial reference geometry as indicated by *C*_tool_^*i*^. After the springback, the formed shape distorted to an error value Δ*L* as seen in Figure 1(1). The reference target shape is then adjusted to Δ*y* = −Δ*L*. The new shape modification field *C*_tool_^*i*+1^ is adjusted directly from the reference product *C*_tool_^*i*^, producing the first compensated die geometry *C*_tool_^*i*+1^ = *C*_tool_^*i*^ + Δ*y* ^*i*^.

This process is basically following the DA method. The new die shape is then used for the next forming as seen in Figure 1(2).

If the springback after the unloading process falls out of tolerance at the first compensation, then the part is bent downwards to the new geometry by applying the inverted internal force *F*_internal_^*i*^ calculated from residual stress (SF algorithm), read as

(1) $$\begin{array}{c} C_{\text{tool}}^{i+1}=C_{\text{tool}}^{i}+F_{\text{internal}}^{i} \end{array}$$

and then sprung forward to the new shape. The deviation should be lower than before and much closer to the reference geometry. If the error is still out of the acceptable tolerance, the compensation process is then continued to new iteration cycle by considering the deviation field (Δ*y* ^*i*+1^) to create a new die surface. The shape deviation field is calculated between the reference geometry to the last springback geometry. The resulting shape modification field needs to be applied following (1) to the last compensation geometry.

The iteration process continues until the formed shape after the springback falls withn the allowable tolerance of the desired shape by implementing an alternate hybrid algorithm of DA and SF. The algorithms are coded in Fortran to do the iteration processes. The internal residual stresses of the formed shape are obtained from the fully formed simulation results. The internal bending stresses are then converted into finite elemental forces as the input load to the HM spring forward phase. The forces are then applied to the finite elements of the springback shape. The result is compared to the original shape to check the shape deviation error.

**Figure 2:**
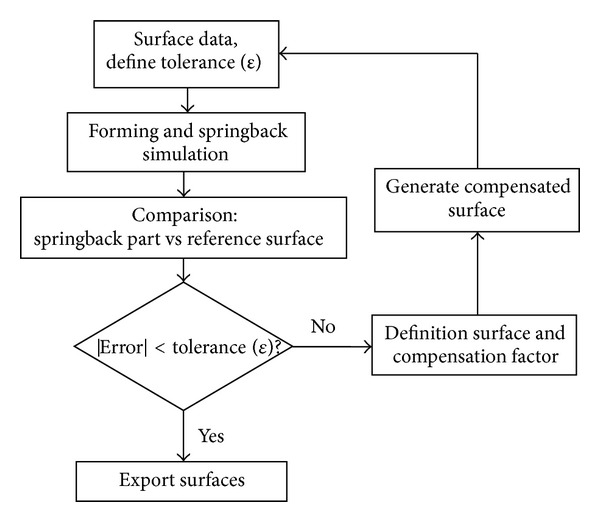
Procedure of die compensation.

To validate the accuracy of the developed hybrid algorithm for die compensation, a compensated die surface generated by hybrid method was tested in Autoform. The springback was then simulated in this software and then compared with the reference shape. To use the die surface of HM in Autoform, the finite elements of the die surfaces should be modified to complete die parts, that is, binder and punch. For this purpose, CATIA was selected to modify and create a surface for springback simulation in Autoform. The HM springback result is then compared with that from Autoform build-in springback compensation module. The tool surfaces are automatically adapted to the new compensated geometry. On the compensation stage, the sprung back shape and the reference geometry are needed. The overall procedure is shown in Figure 2.

The flow of simulations starts from input data of surface die tools consisting of die, punch, binder, and reference surface. The part after springback is compared to the reference geometry for a deviation gap (error) checking. If the error is larger than the allowable tolerance, the die surface needs to be compensated. The generation of compensating surface data is delivered by the software based on DA algorithm. Another simulation is conducted with the new compensated surface until the error is within the tolerance [25].

In this presented research work, the results of HM are compared with those from the stand alone of DA and SF. The comparison results show some critical issues: the maximum error at every iteration cycle, the number of iterations to compensate the die surface, and the total CPU time to converge the compensated die surfaces.

## 3. Numerical Simulation Results

The usage of the hybrid method (HM) algorithm to compensate springback by combining two methods of DA and SF is presented in this section. The results are compared against those from application software Autoform with springback compensation module.

Two models representing two- and three-dimensional problems are used. The two-dimensional (2D) springback model is taken from the U-bending springback problem in NUMISHEET 1993 [26], while the three-dimensional (3D) springback problem is the S-rail benchmark model of NUMISHEET 2008 [27].

### 3.1. Springback of 2D Model

The U-bending problem consists of a set of punch, holder, die, and blank sheet [26]. The thickness of the blank sheet is 0.8 mm with the maximum punch drawing 70 mm at constant speed of 4.3 m/s. The blank holder force varies between 1, 5, 10, 15, 20, and 25 kN. The sheet material is mild steel DC04 having a Young's modulus 210 GPa and Poisson's ratio 0.3. An initial yield stress is 167.9 MPa, the reference stress value *K* is equal to 550 MPa, and the work hardening exponent *n* = 0.223.

The mesh quality in Autoform depends on the sets value of parameters radius penetration, maximum element angle, and maximum displacement angle. The mesh refinement varies from rough, standard, and fine, as shown in Table 1.

**Figure 3:**
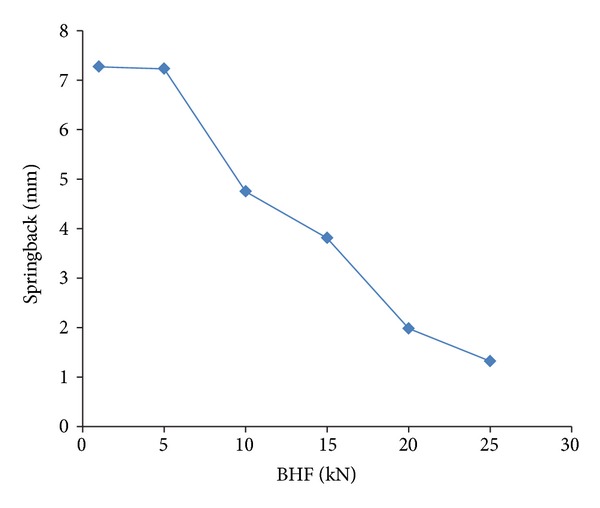
Principal result of springback prediction.

The results of springback in various BHF are shown in Figure 3. The lowest springback deviation recorded in fine mesh is 1.32 mm under the highest BHF of 25 kN. The higher the BHF, the less the springback. Kergen and Jodogne [28] have shown similar BHF trends against springback in determining the minimum BHF for steels. Hishida and Wagoner [29] determined that a quality defect of products formed by stamping processes, such as fracture, wrinkles, and surface distortion, can be suppressed by the selection of the optimum BHF values. For the present 2D model here, the lowest springback can be achieved by applying higher BHF.

**Figure 4:**
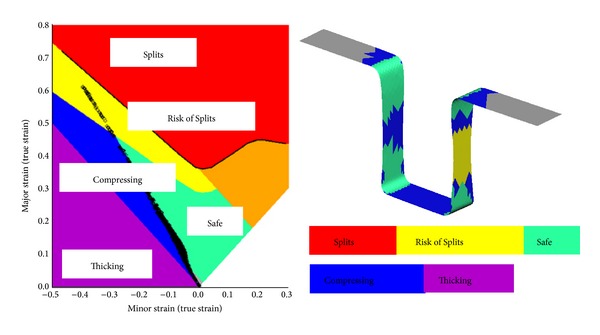
Forming limit diagram under BHF of 27.5 kN.

Investigation of the BHF effect continued with increasing the value to reach the failure in forming simulation and to see the thinning history. The holding forces are extended to 26, 27.2, and 27.5 kN in terms of finding the optimum BHF. Figure 3 and Table 2 show clearly that the high springback deviations (higher than 3 mm) occur when the applied holding forces are at 1, 5, 10, and 15 kN. The risks of failure, thinning, and high strain arise under excessive holding forces at 25, 27, and 27.2 kN. In the range from 25 to 27.5 kN, the simulations fail due to the high risk of splitting and failure. This can be seen from the forming limit diagram shown in Figure 4, as indicated in bright color. The result variable maximum failure is defined as the ratio between the maximum major strain of an element and the major strain on the forming limit curve (FLC) for the same minor strain. Based on the results, the holding force in the range from 20 to 24 kN is suggested as the optimum range of BHF that gives less springback without having a failure risk on the formed sheet.

**Figure 5:**
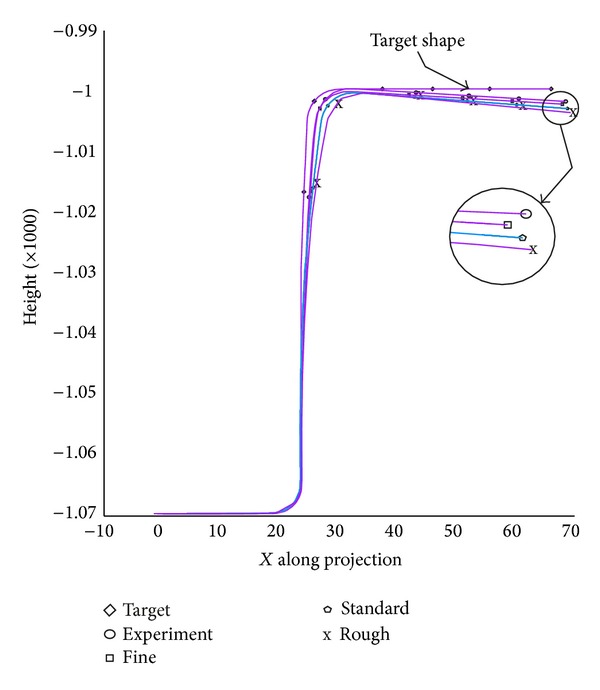
The influence of mesh refinement, BHF 20 kN.

The adaptive refinement mesh type in Autoform affects the results of springback.  Figure 5 shows how element quality affects the springback in the selected holding force 20 kN. The fine meshing shows the closest springback to the experimental result [26]. The highest springback, however, occurs at the flange position. The deviations of other positions of the workpiece are closer to the experimental results.

### 3.2. Compensation of 2D Model

For the compensation testing, the case BHF 20 kN is selected with the fine mesh quality and the friction coefficient 0.3. This coefficient value is for the general lubricated condition in the forming process [11]. In the sheet metal forming, boundary lubrication is the most widely encountered. It is defined as a condition where the solid surfaces are so close. The reference and springback nodes generated by Autoform are modified to the coordinate *x*, *y*, and *z* under text format to suit the HM input format. There are 601 common points being selected at the reference and springback used for the calculation of error and nodes translations.

**Figure 6:**
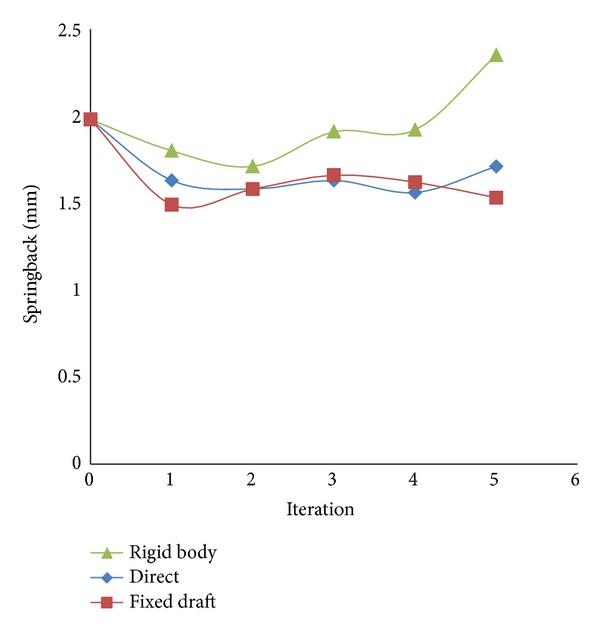
Springback error in every cycle of compensation.

Forming simulation for the second cycle is conducted by using new compensated surfaces which were generated under compensation factor 1.0. The compensation types available in Autoform, direct, fixed, and rigid body, are tried. It can be seen in Table 3 that springback results are higher than the initial springback error 1.98 mm (see Table 2) for rigid body types.  Figure 6 shows the comparison of springback history results for all compensation types. The fixed draft type converges into the smallest springback error 1.53 mm after the fifth iteration.

In rigid body, there are three adjustments to compensate: automatic, automatic *z*-direction, and manual. The entire area defined as a rigid body is compensated relative to the average vector. From the result, this is only effective for a number of iterations not more than two. At the second iteration, the rigid body can reach the lowest springback value 1.58 mm and then deviates more on the next iterations. Direct compensation type is influenced by compensation factors only. The compensation factor is 1.0 in each iteration. This compensation makes translation in every direction (*x*, *y*, *z*) become based on the reference of a springback shape. By using fixed draft type, it allows the compensation of surface regions while maintaining their angle towards working direction. This fixed type gives better result than others and the springback deviation is only 1.53 mm.

**Figure 7:**
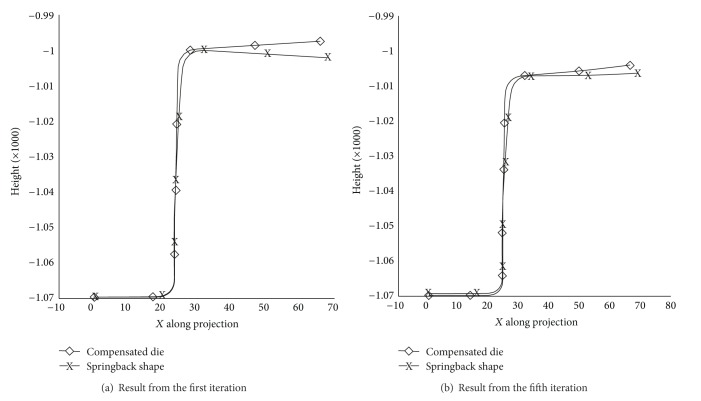
The influence of mesh refinement, BHF 20 kN.

The sectional view of compensated die and springback position can be seen in Figure 7. At the first iteration, the springback position falls below the reference part (Figure 7(a)), whereas the fifth iteration, the unloaded part, stays at the top of reference surface (Figure 7(b)).

**Figure 8:**
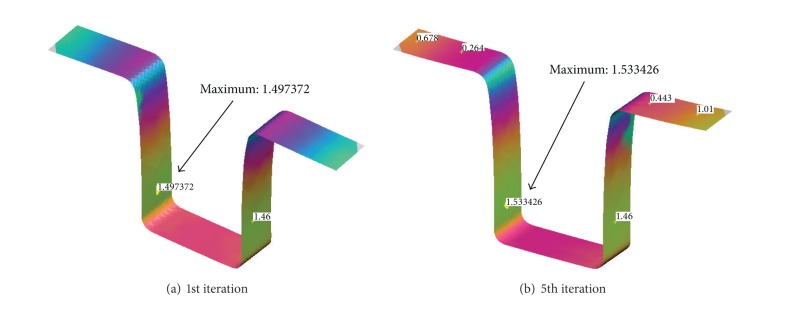
Highest deviation in the sidewall area.

Since the compensation algorithm in Autoform adopts the DA method which considers punch-axis direction, therefore the shape distortion in the sidewall is still significant as is clearly seen in Figure 8.

**Figure 9:**
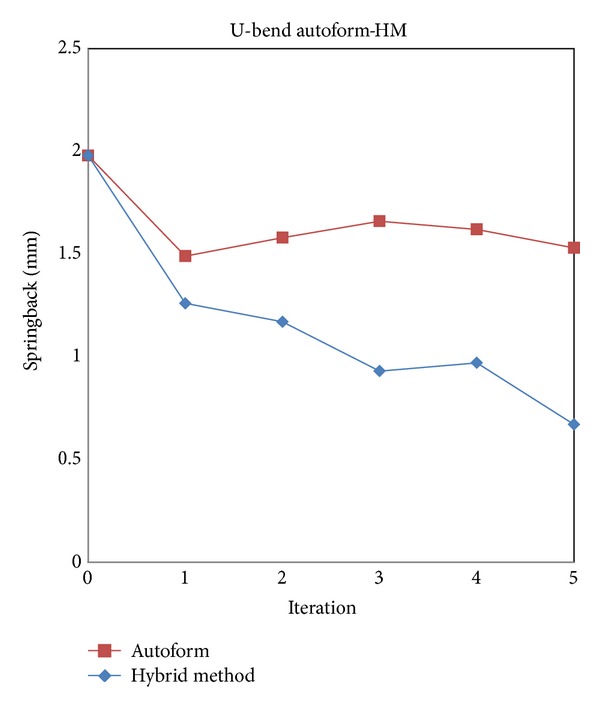
Compensation of U-bending results.

The springback comparison between HM and Autoform for 2D model is shown in Figure 9. It can be seen clearly that HM performs better than Autoform. At the first iteration, the springback can be decreased significantly to 1.26 mm. The error continues to decrease in the second and third iterations, but then it fluctuates in the fourth iteration showing a higher value than the previous one. The springback reduction trend continues to the last (fifth) iteration. The second and third iteration results are getting higher due to the application of SF method. In the first iteration, it gives a higher residual stress than that from the third iteration that will be used in the SF method in the second and fourth iterations. Therefore, the high residual stress has delivered high compensation value too, as shown at the second iteration for 1.17 mm and 0.97 mm at the fourth iteration. The proposed alternate hybrid method of DA and SF has successfully decreased the springback deviation to 0.67 mm (reducing 66% from the initial error), while Autoform decreased the springback to 1.49 mm (reducing 25% from the initial error).

The alternate HM method also improves the accuracy of the formed product in the sidewall. The maximum deviation after the fifth iteration is 0.935 mm, which is lower than that from Autoform result 1.533 mm.

### 3.3. Springback of 3D Model

The benchmark of NUMISHEET 2008 has included an additional problem which is related to springback. In this analysis, the accuracy of springback prediction is presented for three different models, that is, without draw beads, smooth draw beads, and locking draw beads.

The material HX260LAD type of microalloyed steel grades with high yield strength for cold forming has been selected as the blank sheet material. The summary of mechanical properties of the material is shown in Table 4.

Variations of BHF 90, 120, and 150 kN are applied on the blank holder parallel to punch direction. The comparison results use several reference sections of A, B, C, and D [27].

**Figure 10:**
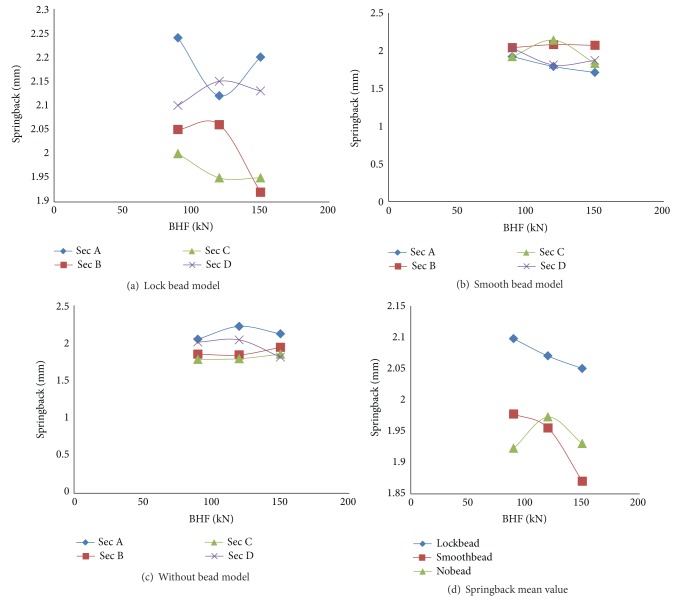
Springback error in sections A, B, C, and D.

The difference of draw bead design in the part geometry has a significant effect on springback. The springback deviation in every section does not show consistent trend when different BHFs are applied. The inconsistent springback results are presented in Figures 10(a), 10(b), and 10(c). In the lock bead model, the increase of BHF from 90 to 120 kN gives higher springback of 2.06 mm and 2.15 mm, in sections B and D, and lower springback in sections A and C of 2.12 mm and 1.95 mm, respectively. When the BHF is applied higher at 150 kN, the springback decreased in sections B and C to 1.92 mm and 1.95 mm, respectively. All of these deviations show that the S-rail is in a twisting mode. In a square cup model where twisting does not occur, Demirci et al. [30] showed the trend of higher BHF to reduce the springback error. In this result, the experimental validation with draw beads is not directly performed. The springback experimental validation however has been conducted in different springback benchmark problems [26, 27] and found that Autoform can predict springback in the range of experimental results.

By looking at the average springback in all sections (Figure 10(d)), the trend of springback in different holding forces can be seen. Both smooth and locked beads show that increasing the holding force can reduce the springback deviation. Under the smooth beads, the springback decreases from 1.97 mm to 1.87 mm when the holder force increased from 90 kN to 150 kN. A similar trend is shown for locked beads. It decreases the springback from 2.1 mm to 2.05 mm. When the drawing process does not use any beads, the tendency of the springback error to the increasing holding force cannot be predicted but fluctuated.

**Figure 11:**
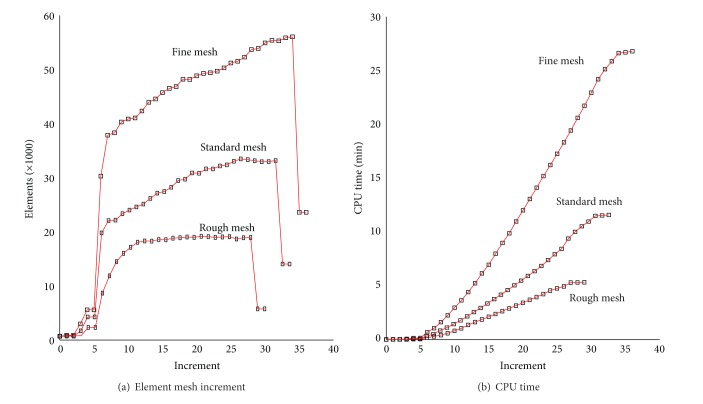
Springback error in sections A, B, C, and D.

In terms of the meshing variation, the recorded mean springback error in standard mesh type (33147 elements) is 1.87 mm, the fined mesh 1.78 mm (56054 elements), and the rough mesh 1.92 mm (17758 elements). The mesh density and the computer CPU (Central Processing Unit) time in Intel i5 processor are shown in Figures 11(a) and 11(b). The total CPU time to finish the simulation for fine mesh type is 26.786 minutes; the standard mesh needs 12.136 minutes and the rough type only needs 5.11 minutes.

The increment number of fines, standard, and rough types are 36, 33, and 29 increments at the end of the simulation.

**Figure 12:**
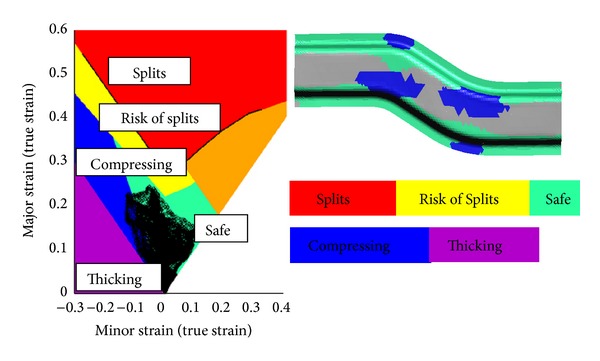
Forming limit diagram of the BHF 150 kN.

The optimum result can be achieved with the combination parameters of holder force 150 kN with smooth draw beads in standard mesh type. The forming limit diagram (FLD) using the mentioned combination is shown in Figure 12. The figure shows that there is no risk of splits and excess thinning when the blank sheet has been fully formed.

### 3.4. Compensation of 3D Model

**Figure 13:**
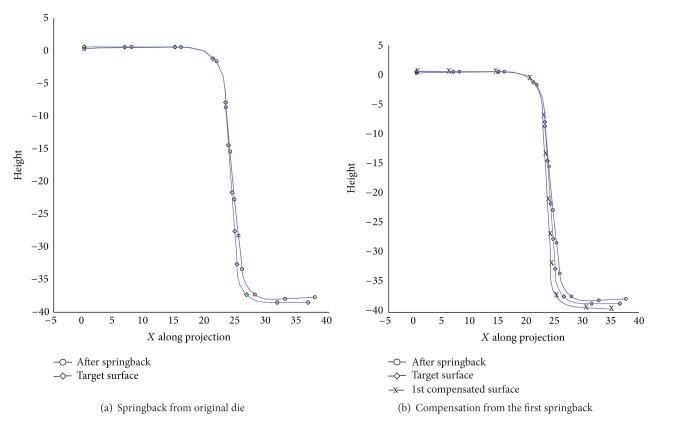
Springback and compensation.

For die compensation, the S-rail model material HX260LAD, BHF 150 kN with smooth beads in standard mesh type, is selected. The mean springback result of this case is 1.87 mm as depicted in Figure 13(a). The reference and springback model of S-rail is converted as point clouds to be used as the input file of HM. The first iteration is conducted by translating the springback nodes following the DA method and then to the inverse of springback (SF algorithm) to get the first compensated die as shown in Figure 13(b).

**Figure 14:**
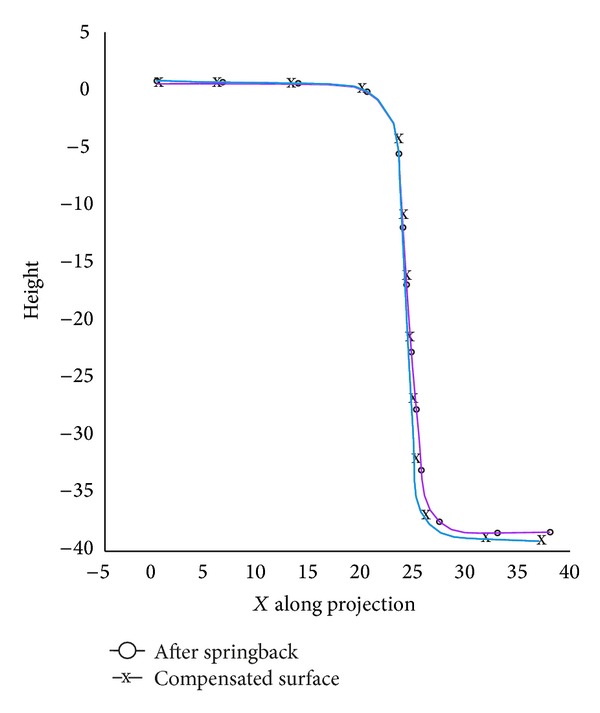
HM die compensation after five cycles.

For convergence control, a tolerance error 1 mm is used. After the fifth cycle, the mean springback can be decreased as low as 0.83 mm and the iteration process terminated (error < 1 mm).  Figure 14 shows the compensated die surface and the springback after fifth iteration.

To use the die surface of HM results in Autoform; the elements must be modified to form the die surface components, that is, binder and punch. CATIA is used to modify and to create surface for springback simulation in Autoform. After springback simulation, the HM result is then compared with the springback from Autoform.

**Figure 15:**
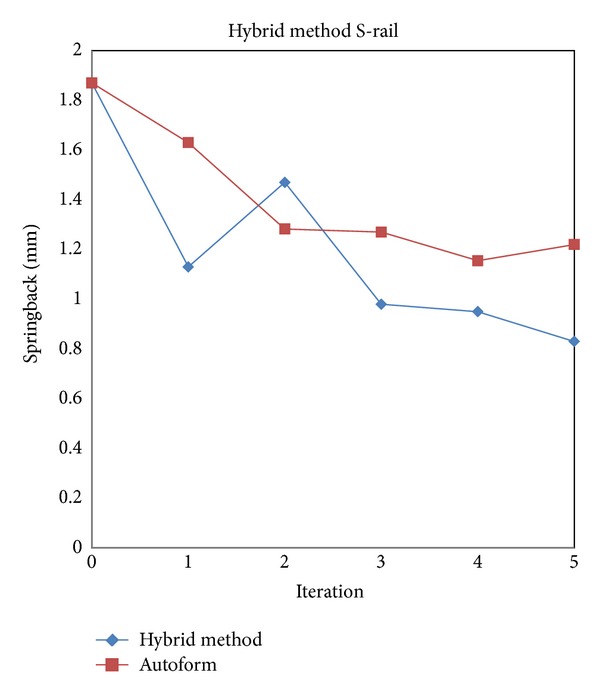
Comparisons between Autoform and HM compensation.

Figure 15 shows the springback compensation history of the alternate HM method compared with Autoform. The first iteration of HM has decreased the springback error to 1.13 mm (39% reduction from initial value). With the alternate DA and SF, after the fifth iteration, the springback deviation can be reduced by 55% from the initial error value from 1.87 mm to 0.83 mm while Autoform can only reduce to 1.23 mm or 34% reduction.

The fluctuation of HM shown in Figure 15 is due to the switching method from DA to SF in the first time. In the next iteration cycle, the shape is in a stable condition and can converge to reduce the springback deviation.

## 4. Discussion

In die compensation, the die surface results rely on springback analysis and the deflection history of elements under the bending and unbending processes.

Deformation theory could be used in prediction instead of flow theory. Accurate data and parameters will require more complicated optimization to accommodate deformation history. Therefore, the springback analysis accuracy was influenced by many factors.

The use of appropriate material model in springback analysis is one factor to improve the accuracy because the correct material can provide an accurate stress state at the end of the forming stage. It is useful if the material model is based on the initial yielding and hardening parameters obtained from the average of multiple experiments. The accuracy of springback prediction is also influenced by the coefficient of friction between the blank sheet and the die surfaces.

The use of combination between optimum blank holder force and draw beads will reduce the springback deviation. For complicated three-dimensional model where the springback is not as simple as bending problem but involving twisting mode, finding the optimum drawing parameters is not straight forward, but a simulation series is required to find the best configuration for a minimum springback.

Springback distortion also depends on the bending moment which in turn depends on the stress distribution through sheet thickness. Shell elements require numerical integration of stress and strain distribution. The largest of integration points will improve the accuracy, but consequently is more time consuming.

In these works, the HM has been coded in Fortran mainly to enable the alternate methods of DA and SF. The displacement adjustment (DA) strategy is intended to generate compensated nodes by translating the nodes in the opposite direction to the springback. The magnitude of the vector translations calculated from the origin and springback position are distributed in all directions Δ*y*, Δ*x*, and Δ*z* corresponding to the axes of *x*, *y*, and *z*.

The strategy to speed up the springback reduction is by using a large compensation factor to the translation vectors which later copied to the die surfaces. This reduces the springback error significantly in the first cycle (see Figure 15).

In the subsequent process, the applied load force in spring forward algorithm is obtained from the internal stress at fully loaded phase. The stresses are converted to inverted load forces.

## 5. Conclusion

In this research, two different methods, displacement adjustment (DA) and spring forward (SF), are joined in alternate manner to compensate the die tools to minimize springback error called hybrid method (HM). When it is used in one algorithm, there are advantages to converge faster and abilities to compensate in all sides. A new approach in springback accommodation using the alternate hybrid method (HM) has been tested in two- and three-dimensional springback problem models. The results show that in two-dimensional models, it can reduce the springback up to 66% in after five iteration cycles and in three-dimensional 55%. In the comparison result with Autoform, the HM method shows better performance while the Autoform can reduce 22% and 35% only for two- and three-dimensional models. The HM is an alternate method to reduce springback error based on die tool compensation. This is applicable for sheet metal forming on stamping process having lower and upper dies.

The implication of this research is that the approach of accommodating springback can be further extended to the implementation of HM in user friendly application software. In this work, the proposed HM approach works on finite element nodes and uses an external CAD program to modify them to point clouds before redrawing them as the die parts. In the future study, an automatic implementation of HM should be ported in a finite element forming simulation program so that the implementation will be easier and user friendly.

## Figures and Tables

**Table 1:** Properties of mesh refinement.

Accuracy	Radius penetration (mm)	Maximum element angle (deg)	Maximum displacement angle (deg)
Rough	0.32	45	3.2
Standard	0.22	30	2.2
Fine	0.16	22.5	1.6

**Table 2:** BHF effect in springback, thinning, strain, and failure.

BHF (kN)	Springback (mm)	Thinning (%)	Plastic strain	Failure
1	7.27	0.02	0.07	0.08
5	7.23	0.02	0.07	0.07
10	4.75	0.04	0.08	0.13
15	3.81	0.04	0.09	0.19
20	1.98	0.06	0.12	0.28
25	1.32	0.08	0.16	0.4
26	1.31	0.08	0.20	0.44
27	1.31	0.11	0.30	0.6
27.2	1.31	0.14	0.40	0.7
27.5	1.36	0.20	0.60	1.0

**Table 3:** Springback error after the fifth compensation, BHF 20 kN.

Type	Compensation factor	Working direction	Springback
Direct	1.0	*x*, *y*, *z*	1.71
Fixed draft	1.0	*z*	1.53
Rigid body	1.0	*z*	2.35

**Table 4:** Summary of mechanical properties HX260LAD.

Orient.	Thickness (mm)	Yield stress (MPa)	U.T.S (MPa)	Uniform elongation %	*r*-value
L	1.00	394.3	463.7	16.4	0.581
T	1.00	427.7	466.0	17.5	1.013
D	1.00	395.3	447.0	17.0	1.166

Mean	1.00	405.8	458.9	16.9	0.981
